# Impact of medication therapy management (MTM) service model on multi-morbidity (MMD) patients with hypertension: a pilot RCT

**DOI:** 10.1186/s12877-023-03725-4

**Published:** 2023-01-06

**Authors:** Na Li, Jin-fang Song, Ming-zhu Zhang, Xiao-min Lv, Hui-lian Hua, Yi-ling Chang

**Affiliations:** 1 Department of Pharmacy, Taizhou People’s Hospital, No.366, Taihu Road, Taizhou City, 225300 Jiangsu Province China; 2 Department of Clinical Pharmacy, Affiliated Hospital of Jiangnan University, Wuxi, Jiangsu Province China; 3 Jiangsu Key Laboratory of New Drug Research and Clinical Pharmacy, Xuzhou Medical University, Xuzhou, Jiangsu Province China; 4 Shandong Provincial Third Hospital, Jinan, Shandong Province China

**Keywords:** Medication therapy management (MTM), Multi-morbidity (MMD), Polypharmacy, Clinical pharmacist

## Abstract

**Background:**

This study explored the impact of MTM service on MMD patients with hypertension.

**Methods:**

A total of 120 MMD inpatients from September to November 2019 were received and randomly divided into intervention group and control group. General services for noninfectious chronic diseases were given to the control group, while a standard MTM service was given to the intervention group. Patients’ blood pressure, EQ-5D utility value, readmission rate, drug-related problems, and average daily medication therapy cost were compared between the two groups and within the groups. This was done at the initial admission phase and in the first, third, sixth, and twelfth months after discharge.

**Results:**

The intervention group had significantly lower blood pressure and average daily medication therapy cost 12 months after discharge compared to the control group (systolic blood pressure: *P* = 0.023, diastolic blood pressure: *P* < 0.001, average daily medication therapy cost: *P* = 0.049); the number of DRPs decreased in both groups 12 months after discharge; the number of DRPs solved in the intervention group in the third, sixth and twelfth months after discharge were statistically higher compared with that in the control group (*P* = 0.013, *P* = 0.012, *P* = 0.001); there was no significant difference in the EQ-5D utility value and readmission rate between the two groups (*P* > 0.05).

**Conclusions:**

MTM implementation in MMD patients can improve health outcomes and reduce healthcare-related costs among MMD patients.

**Trial registration:**

Chinese Clinical Trial Register ChiCTR2200065111, date of registration: October 28, 2022.

**Supplementary Information:**

The online version contains supplementary material available at 10.1186/s12877-023-03725-4.

## Background

Noninfectious chronic diseases (NCDs) refer to diseases with slow pathological changes or which cannot be cured in a short time, such as hypertension, diabetes, and coronary heart disease [1]. At present, NCDs may cause high mortality, morbidity, and disability, affecting the health of Chinese people [2]. NCDs often have many etiological factors during the long course of the disease. Thus, patients are often trapped in a disease “cumulation” state resulting from multiple causes. Further, people suffer from multi-morbidity (MMD), which means simultaneously suffering from two or more NCDs [3]. According to many studies, the prevalence rate of MMD in middle-aged and older adults is 57–74%, and MMD has become one of the most serious public health threats in China [4–6]. Patients’ quality of life is affected due to the impact of MMD on social, psychological, physiological, spiritual, and economic factors [7]. According to Report on Cardiovascular Health and Diseases Burden in China: An Updated Summary of 2020, vascular disease is the leading cause of death [8]. As one of the most common NCDs with a high incidence and a long course of the disease, hypertension is the main risk factor for inducing cardiovascular diseases [9]. It is also the main cause of death in patients with cardiovascular and cerebrovascular diseases in China. The number of patients with hypertension in China has reached 245 million [8].

MMD patients frequently need polypharmacy. Polypharmacy usually refers to the simultaneous use of five or more drugs, including over-the-counter and prescription drugs, Chinese herbal medicines, and other health products [10]. With the growing population of aged people and the high incidence of MMD, multi-drug usage is also increasing yearly [11]. The rate of polypharmacy in elderly MMD patients in communities of China is 33.1–75.3% [12], and the rate of polypharmacy in hospital inpatients is 48.0–95.7% [13]. Further, polypharmacy is an important method to control and treat MMD. However, adverse consequences may also occur, including increased drug-related problems (DRPs), poor patient compliance, increased readmission rate, and prolonged hospitalization time [14].

Medication therapy management (MTM) refers to the process in which a series of professional services such as medication education, treatment consultation, and guidance on the administration of therapeutics are provided by pharmacists and pharmaceutical professionals with technical know-how [15]. This is done to prevent medication errors, and train patients to conduct self-medication management to improve the curative effect [16]. The MTM focuses on five core elements such as medication therapy review (MTR), personal medication record, medication action plan, intervention and referral, documentation and follow-up, and solving patients’ DRPs. The challenges associated with MMD and polypharmacy have attracted global attention. However, multi-drug therapy patients are more likely to get help from MTM services [17].

This study will establish the MTM model for MMD patients with hypertension. This model will increase understanding of therapeutic drugs, reduce DRPs, reduce their financial burden, and actively convey health consciousness and a healthy lifestyle to patients. Meanwhile, pharmacists will be assisted in improving their pharmaceutical services and MMD management capabilities, strengthening their communication with patients, improving their service enthusiasm, and reflecting on their professional value.

## Materials and methods

### Objectives

This study aimed to explore the impact of a 12-month pharmacist-based MTM service mode on MMD patients with hypertension.

### Participants

One hundred twenty patients with hypertension who were hospitalized in Taizhou People’s Hospital, Taizhou, China, from September to December 2019, were selected as participants. This study was a randomized controlled trial, which was divided into intervention group and control group using a computer-generated random sequence. Further, the participants were subjected to detailed interviews and rigorous evaluations.

The patients’ systolic blood pressure was taken as the observation index. If the intervention group reduced their systolic blood pressure by 5.86 mmHg more than the control group did, then the drug was considered to have application value. According to a literature review and preliminary experimental results, the standard deviation of systolic blood pressure reduction in the control group andintervention group was 10.70 mmHg and 8.11 mmHg, respectively. Moreover, according to the 1:1 parallel control design, the unilateral test was set as unilateral α = 0.025, and the degree of assurance was 80%. Using PASS 15 software, the sample size of the control group and the intervention group was calculated to be 43 cases, and a total of at least 86 cases were included in this study. Considering the possible shedding rate and the period of our study, 60 cases in each group was determined as the sample to be included in this study. At the end of the study, 9 participants in the intervention group and 10 in the control group were lost to follow-up. Participants who were lost to follow-up were eliminated from the study. Finally, 51 participants in the intervention group and 50 in the control group completed the follow-up for 12 months, with complete data collected for analysis. The overall drop-out rate was 15.8%. Drop-out rates were 16.7% and 15.0% among control and intervention group participants, respectively. No statistical association was observed between group assignment and study drop-outs, as shown in the additional file 1.

Inclusion criteria were (1) age 45–80 years; (2) suffering from more than two NCDs with hypertension which was diagnosed in conformity with the standard of primary hypertension specified in the Guidance for Hypertension Prevention and Control Management at the National Grassroots Level (Version 2020); (3) using more than five drugs; and (4) informed consent to participate in this survey, conscious and able to communicate effectively. The main exclusion criteria included (1) with psychological and mental illnesses or other mental health conditionsrequiring medication; (2) being diagnosed with New York Heart Association Class III or IV heart failure; (3) with critical illnesses such as severe hepatic impairment, renal insufficiency, and malignant tumors, who were unable to withstand the process and effects of the evaluation; (4) being unreachable at the time of the first telephone follow-up visit and having no other contact information. Patients who died or without complete clinical data were also excluded.

### Study design

Eligible MMD patients with hypertension were invited to participate in the study. Written informed consent was obtained from each participant before the study. The study was registered at the Chinese Clinical Trial Registry (registration number: ChiCTR2200065111, date of registration: 28/10/2022).

The clinical pharmacists providing MTM services reviewed the assessment method of blood pressure used at the clinic with the medical director to ensure consistency of the measurements between the intervention group and the control group before study initiation. All inpatients collected blood pressure data using the yuyue brand (U30 type) arm electronic sphygmomanometer. Before taking antihypertensive drugs on the morning of the measurement day, the patient was placed in a sitting or lying position in a calm state and measured by the clinical pharmacists. The mean value of three consecutive blood pressure measurements was taken.

Following the five core elements of the MTM service model, the clinical pharmacists in Taizhou People’s Hospital conducted the MTR at the initial phase of admission, integrated all the drugs used by patients, and collated and completed patients’ drug records (including medication during hospitalization). Meanwhile, a face-to-face questionnaire was conducted to collect patients’ demographics (age, education level, pre-retirement occupation, living structure, family monthly income), NCDs status (types of NCDs, past allergy history, medical history, and current medication), health-related influencing factors (smoking, drinking, BMI, and family medical history, comorbidities), patients’ clinical indicators (required parameter: blood pressure; optional parameters according to comorbidities: heart rate, alanine aminotransferase, aspartate aminotransferase, urea nitrogen, serum creatinine, estimated glomerular filtration rate, fasting blood glucose, 2-h postprandial blood glucose, glycosylated hemoglobin, total cholesterol, triglyceride, low-density lipoprotein cholesterol, high-density lipoproteincholesterol, uric acid, etc.), average daily medication therapy cost, EQ-5D utility value, and DRPs status. After completing the questionnaire, the pharmacists checked each patient’s medication history in the information system of Taizhou People’s Hospital (HIS).

**Fig. 1:**
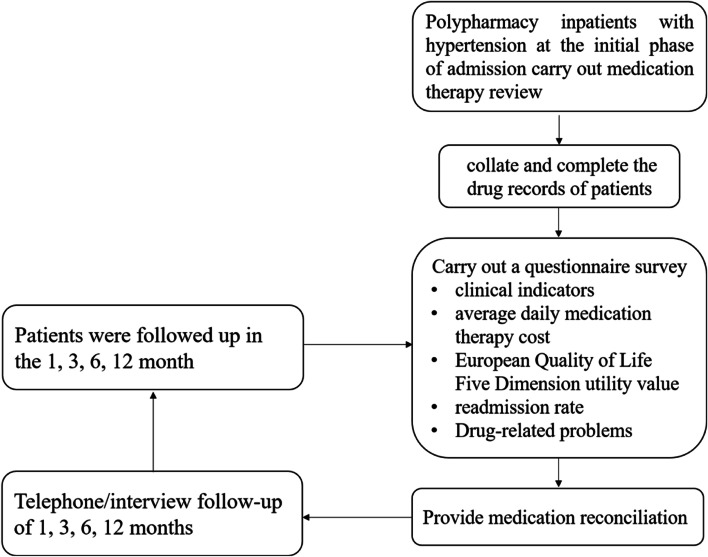
Service flow chart of the intervention group

Clinical pharmacists from Taizhou People’s Hospital followed up with the patients via telephone and face-to-face interviews in the first, third, sixth, and twelfth months after discharge. The follow-up was on DRPs inquiry and medication education. The control group received general pharmaceutical care. The follow-up content for the control group included clinical indicators, names, and dosages of drugs used, EQ-5D utility value, hospitalization, and DRPs status. The follow-up content of the intervention group was based on the follow-up contents of the control group; the medication reconciliation was carried out for the patients with multi-drug usage, as shown in Fig. 1. Evaluation indexes were collected for both groups during hospitalization and follow-up at 1, 3, 6, and 12 months. During the follow-up, comorbidities (included mainly coronary heart disease, diabetes mellitus, heart failure, hyperlipidemia, renal insufficiency, renal artery stenoses) were tracked mainly by clinical indicators and medication. Once the clinical indicators were seriously abnormal, such as severe liver function damage, renal insufficiency or other circumstances that meet the exclusion criteria, they would be excluded from this study.

### Observation indicators

(1) Observation of the drug use by patients: the blood pressure value at the initial phase of admission and in the first, third, sixth, and twelfth months after discharge were recorded and compared. The pharmacists also trained patients to use the electronic arm sphygmomanometer correctly. (2) The EQ-5D utility value was obtained using the European Quality of Life Five Dimension Five Level Scale Questionnaire, known as EQ-5D-5L. The scale comprises five dimensions: mobility, self-care, usual activities, pain/discomfort, and anxiety/depression. Further, each dimension has five levels: no problems, slight problems, moderate problems, severe problems, and extreme problems. The Chinese EQ-5D-5L score conversion system table was used [18]. Based on the highest utility value of one, according to the evaluation level of each dimension, the corresponding coefficient value was deducted to obtain the utility value of the patient, as shown in Table S1. (3) Average daily medication therapy cost: the cumulative cost of daily medication. The clinical pharmacists searched the hospital catalog used by patients in the information system of Taizhou People’s Hospital (HIS), to determine the costs of the drugs. For the drugs that could not be found in the hospital catalog, the pharmacists searched the Yaozhi Medication App. Finally, the pharmacists calculated the daily cost of each drug based on the patient’s usage and dosage. (4) Readmission rate: the ratio of the number of patients readmitted in each group to the total number of patients. (5) Analysis of DRPs: the Strand classification system of Professor Hepler and Strand [19] was used to divide the DRPs into four categories and seven problems [20]. The number was recorded with one rule as one DRPs, as shown in Table S2.

### Statistical analyses

The SPSS software version 23.0 was used for statistical analysis. The continuous variables were shown as mean ± standard deviation, and independent-samples t test was used for comparison between two groups. Repeated measures data were analyzed using ANOVA followed by post hoc analysis with Bonferroni adjustment for multiple comparisons. Count data were expressed as frequencies (%) and compared by Pearson's Chi-square test. The power calculator software PASS used for statistical power calculations. A value of *P* < 0.05 was considered statistically significant.

## Results

### Baseline characteristics

The baseline characteristics of 101 enrolled patients are shown in Table 1. The average age was about 64 years, and 54.5% (55 of 101) patients were men. Only 5% (5 of 101) of the patients lived alone, and 65.3% (66 of 101) had a junior high school education or higher. 30.7% (31 of 101) of the patients smoked and 15.8% (16 of 101) drank alcohol. More than half of the patients had a BMI over 24. The family monthly income of the intervention group was 7188.24 ± 4894.87, and that of the control group was 12,250.00 ± 4780.47. Comorbidities included cardiovascular disease, diabetes, dyslipidemia, and chronic kidney disease. Based on the comparison between the intervention and control groups, there was no significant difference in the basic characteristics such as gender, age, living status, education level, pre-retirement occupation, family monthly income, smoking habit, drinking practice, and BMI (*P* > 0.05).

**Table 1:** Comparison of basic characteristics between two groups

Variable	Intervention group (*n* = 51), n(%)	Control group (*n* = 50), n(%)	χ^2^ value	*P* value
**Gender**			6.194	0.091
Male	34 (66.7)	21 (42.0)		
Female	17 (33.3)	29 (58.0)		
**Age (years)**	64.06 ± 9.43	63.42 ± 9.06	6.229	0.596
45–49	0 (0.0)	4 (8.0)		
50–59	17 (33.3)	12 (24.0)		
60–69	17 (33.3)	20 (40.0)		
70–79	16 (31.4)	14 (28.0)		
≥ 80	1 (2.0)	0 (0.0)		
**Living arrangement**			0.232	0.34
Living alone	2 (3.9)	3 (6.0)		
Not living alone	49 (96.1)	47 (94.0)		
**Education level**			1.819	0.992
Primary school and below	15 (29.4)	20 (40.0)		
Junior high school	19 (37.3)	18 (36.0)		
Senior high school	14 (27.4)	9 (18.0)		
University and above	3 (5.9)	3 (6.0)		
**Pre-retirement occupation**			6.257	0.064
Civil servant	2 (3.9)	0 (0.0)		
Staff of enterprises and institutions	34 (66.7)	34 (68.0)		
Farmer	7 (13.8)	13 (26.0)		
Freelancer	4 (7.8)	1 (2.0)		
Unemployed	4 (7.8)	2 (4.0)		
**Family monthly income (RMB)**	7188.24 ± 4894.87	12,250.00 ± 4780.47	29.074	0.965
< 5000	20 (39.2)	0 (0.0)		
5000–10,000	19 (37.3)	24 (46.0)		
10,000–15,000	5 (9.8)	11 (24.0)		
15,000–20,000	7 (13.7)	9 (18.0)		
≥ 20,000	0 (0.0)	6 (12.0)		
**Smoking or not**			0.983	0.054
Yes	19 (37.3)	12 (24.0)		
No	32 (62.7)	38 (76.0)		
**Drinking or not**			0.252	0.32
Yes	9 (17.6)	7 (14.0)		
No	42 (82.4)	43 (86.0)		
**BMI(kg/m**^**2**^**)**	24.55 ± 3.49	24.61 ± 3.37	0.365	0.494
< 18.5	2 (3.9)	1 (2.0)		
18.5–23.9	21 (41.2)	20 (40.0)		
≥ 24	28 (54.9)	29 (58.0)		
Comorbidities			3.034	0.699
coronary heart disease	29	28		
diabetes mellitus	23	21		
heart failure	18	20		
hyperlipidemia	28	17		
renal insufficiency	21	17		
renal artery stenoses	10	13		

### Systolic and diastolic blood pressure of patients

In the intervention group, comparing the systolic and diastolic blood pressure in the sixth months after discharge and at the initial phase of admission, it differed with a statistical significance (*P* < 0.05). However, in the control group, only systolic blood pressure was significantly lower than the initial phase of admission (*P* < 0.05).

**Fig. 2:**
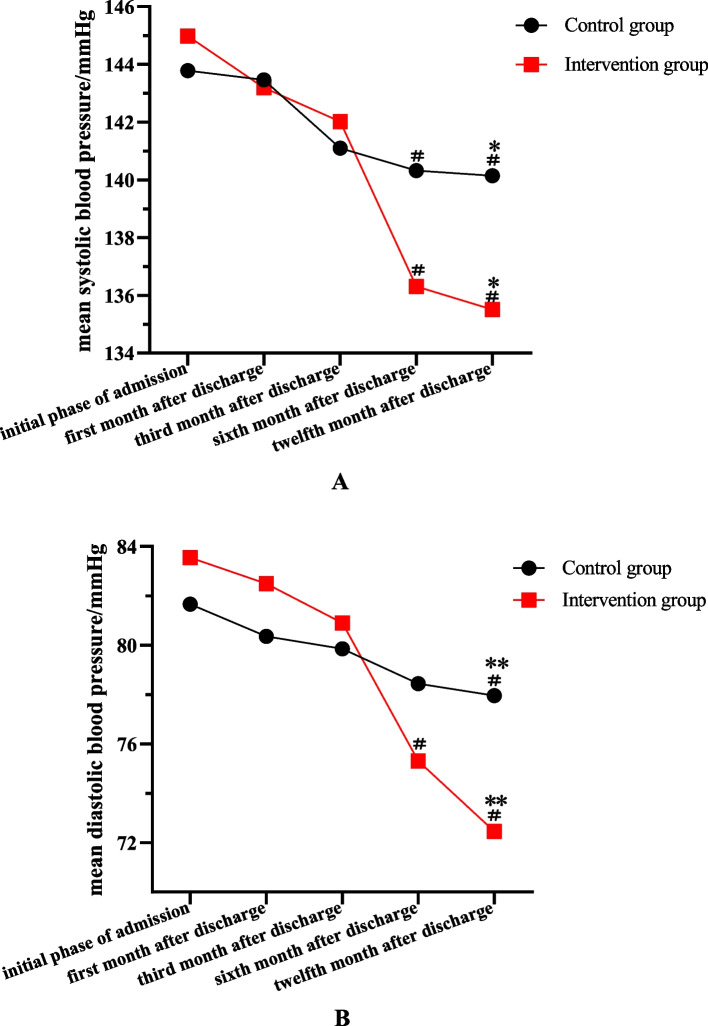
Comparison of systolic blood pressure and diastolic blood pressure between two groups. **A** Mean systolic blood pressure. **B** Mean diastolic blood pressure. Mean systolic blood pressure and mean diastolic blood pressure were compared with that of the group at the initial phase of admission, ^#^*P* < 0.05; Mean systolic blood pressure and mean diastolic blood pressure were compared between the two groups, ^*^P < 0.05; ^*^^*^P < 0.001

In the twelfth months after discharge, the mean intervention group systolic blood pressure decreased by 9.47 mm Hg compared with the initial phase of admission, and the control group decreased by 3.64 mm Hg. The difference of the mean systolic blood pressure between the control and the intervention group (6.56 mm Hg) was statistically significant (*P* = 0.023). Compared with the initial phase of admission, the mean diastolic blood pressure of the intervention group and the control group decreased by 11.1 mm Hg and 3.7 mm Hg, respectively. The mean diastolic blood pressure change of the control group and the intervention group (7.4 mm Hg) was significantly different (*P* < 0.001). The results are shown in Table 2 and Fig. 2.

**Table 2:** Comparison of systolic blood pressure and diastolic blood pressure between two groups (mmHg)

Group	Systolic blood pressure					Diastolic blood pressure				
	At the initial phase of admission	In the first month after discharge	In the third month after discharge	In the sixth month after discharge	In the twelfth month after discharge	At the initial phase of admission	In the first month after discharge	In the third month after discharge	In the sixth month after discharge	In the twelfth month after discharge
Intervention group (*n* = 51)	144.98 ± 14.78	143.20 ± 12.75	142.02 ± 13.27	136.31 ± 11.82^a^	135.51 ± 9.99^ab^	83.55 ± 11.21	82.49 ± 11.27	80.90 ± 11.13	75.31 ± 9.11^a^	72.45 ± 6.76^ab^
Control group (*n* = 50)	143.78 ± 13.95	143.46 ± 12.84	141.10 ± 13.29	140.32 ± 14.71^a^	140.14 ± 10.23 ^ab^	81.66 ± 12.39	80.36 ± 10.74	79.86 ± 10.07	78.44 ± 10.45	77.96 ± 8.67^ab^
^b^*P* value	0.676	0.918	0.729	0.134	0.023	0.424	0.333	0.623	0.112	< 0.001

Systolic blood pressure and diastolic blood pressure were compared with that of the group at the initial phase of admission, ^a^*P* < 0.05;

systolic blood pressure and diastolic blood pressure were compared between the two groups, ^b^*P* < 0.05

### EQ-5D utility value

The two groups had higher EQ-5D utility values in the first, third, sixth, and twelfth months after discharge compared with that at the initial phase of admission, with a statistical significance (*P* < 0.05), but no significant difference was found between the groups (*P* > *0.05*), as shown in Table 3.

**Table 3:** EQ-5D utility value between two groups

Group	At the initial phase of admission	In the first month after discharge	In the third month after discharge	In the sixth month after discharge	In the twelfth month after discharge
Intervention group (*n* = 51)	0.81 ± 0.13	0.84 ± 0.09^a^	0.86 ± 0.09^a^	0.91 ± 0.07^a^	0.95 ± 0.06^a^
Control group (*n* = 50)	0.78 ± 0.12	0.80 ± 0.10^a^	0.85 ± 0.08^a^	0.89 ± 0.08^a^	0.96 ± 0.04^a^
^b^*P* value	0.191	0.071	0.355	0.394	0.533

EQ-5D utility value was compared with that of the group at the initial phase of admission, ^a^*P* < 0.05; EQ-5D utility value was compared between the two groups, ^b^*P* < 0.05

### Average daily medication therapy cost

The average daily medication therapy cost of two groups in the first, third, sixth, and twelfth months after discharge decreased compared with that at the initial admission phase. In the intervention group, the average daily medication therapy cost in the first, third, sixth, and twelfth months after discharge was significantly lower than in the initial admission phase (*P* < 0.05). During the follow-up in the twelfth month after discharge, compared with that of the control group, the average daily medication therapy cost of the intervention group was significantly lower (*P* < 0.05), as shown in Table 4.

**Table 4:** Average daily medication therapy cost between two groups (RMB1)

Group	At the initial phase of admission	In the first month after discharge	In the third month after discharge	In the sixth month after discharge	In the twelfth month after discharge
Intervention group (*n* = 51)	50.58 ± 26.86	45.50 ± 23.56^a^	42.83 ± 22.89^a^	39.17 ± 21.51^a^	35.43 ± 17.83^ab^
Control group (*n* = 50)	48.90 ± 30.84	46.07 ± 30.46	46.50 ± 30.58	45.75 ± 30.51	46.03 ± 33.13^b^
^b^*P* value	0.771	0.916	0.497	0.214	0.049

Average daily medication therapy cost was compared with that of the group at the initial phase of admission, ^a^*P* < 0.05; average daily medication therapy cost was compared between the two groups, ^b^*P* < 0.05

### Readmission rate

The readmission rate of the intervention group in the sixth month after discharge was 7.84%, and 18.0% in the control group. After a 12-month follow-up, the readmission rate in the intervention group was 13.73% and 24% in the control group. Compared with the control group in the sixth, and twelfth months after discharge, the intervention group was lower but with no statistical significance (*P* > 0.05).

### DRPs identified in MTM

There was no significant difference in the number of DRPs between the two groups (*P* > 0.05) at the initial phase of admission. With the follow-up, the number of DRPs solved in the intervention group in the third, sixth and twelfth months after discharge were statistically higher compared with that in the control group (*P* < 0.05). The patients in the intervention group had a maximum of eight DRPs at the initial phase of admission, and a maximum of seven DRPs was reduced after a 12-month follow-up; however, a maximum of three DRPs was reduced for the patients in the control group, and DRPs increased in five patients. During the drug use, there were one or more DRPs. As shown in Table 5.

**Table 5:** Number of DRPs between two groups

Group	the initial phase of admission	*P* value	the first month after discharge—the initial phase of admission	*P* value	the third month after discharge—the initial phase of admission	*P* value	the sixth month after discharge—the initial phase of admission	*P* value	the twelfth month after discharge—the initial phase of admission	*P* value
Intervention group (*n* = 51)	1.67 ± 1.37	0.959	-0.61 ± 1.06	0.084	-0.78 ± 0.99^b^	0.013	-0.90 ± 1.12^b^	0.012	-1.25 ± 1.07^b^	0.001
Min, Max	0, 8		-4, 2		-5, 1		-6, 1		-6, 0	
Control group (*n* = 50)	1.68 ± 1.24		-0.28 ± 0.81		-0.32 ± 0.84^b^		-0.40 ± 0.83^b^		-0.56 ± 0.86^b^	
Min, Max	0, 6		-2, 1		-2, 2		-3, 1		-3, 1	

Number of DRPs was compared between the two groups, ^b^*P* < 0.05

The most common DRPs of the two groups related to non-adherence and adverse drug reaction. After a 12-month follow-up, the number of DRPs in the intervention group decreased from 85 at the initial phase of admission to 21, 75.3% DRPs solved. Further, the number of DRPs in the control group decreased from 84 at the initial phase of admission to 56. MTM services improved 81.0% of non-adherence and 72.2% of adverse drug reactions. More details and outcome of DRPs identified among the two groups are listed in Table 6.

**Table 6:** Types of DRPs identified and level of intervention for each DRPs between two groups

DRPs Classification		At the initial phase of admission		In the twelfth month after discharge		Confirmed as resolved, n (%)	
		Intervention group	Control group	Intervention group	Control group	Intervention group	Control group
		(*n* = 51)	(*n* = 50)	(*n* = 51)	(*n* = 50)	(*n* = 51)	(*n* = 50)
Indication	Drug without indication	16(18.8)	13(15.5)	5(23.8)	10(17.9)	11(17.2)	3(10.7)
	Untreated indication	6(7.1)	8(9.5)	1(4.8)	5(8.9)	5(7.8)	3(10.7)
Effectiveness	Different drugs need to be selected	13(15.3)	11(13.1)	5(23.8)	8(14.3)	8(12.5)	3(10.7)
	Underdose	4(4.7)	3(3.6)	0(0.0)	1(1.8)	4(6.3)	2(7.1)
Safety	Adverse drug reaction	18(21.2)	22(26.2)	5(23.8)	16(28.6)	13(20.3)	6(21.4)
	Overdose	7(8.2)	8(9.5)	1(4.8)	3(5.4)	6(9.4)	5(17.9)
Compliance	Non-adherence	21(24.7)	19(22.6)	4(19.0)	13(23.2)	17(26.6)	6(21.4)
Total		85(100.0)	84(100.0)	21(100.0)	56(100.0)	64(100.0)	28(100.0)

## Discussion

In this study, MTM was implemented in middle-aged and elderly MMD patients with hypertension to observe the impact of MTM on blood pressure, EQ-5D scale, average daily medication therapy cost, readmission rate, and number of DRPs.

We found that diastolic and systolic blood pressure of the intervention group in the sixth and twelfth months after discharge were significantly lower than that at the initial phase of admission (*P* < 0.05). Moreover, the control of diastolic and systolic blood pressure in the intervention group was significantly better than in the control group in the twelfth month after discharge (*P* < 0.05). During the MMD management, MTM service may have helped to control the blood pressure in patients with hypertension, which is beneficial to the treatment of hypertension in longer periods of discharge and can be regarded as one of the key tasks of pharmacists.

NCDs are the main ailments threatening health and deteriorating the quality of life. Therefore, the pharmaceutical service should also focus on improving the patients’ quality of life [21]. In this study, after a 12-month follow-up of the two groups of patients, the EQ-5D utility value increased compared with that at the initial admission phase. However, there was no statistical difference between the two groups. From the results of this study, the MTM service was not confirmed to be better in improving the utility value than the general pharmaceutical service. These results may be because the MMD patients selected in the current study had hypertension and the symptoms of hypertension often vary from person to person; most of them are asymptomatic or not obvious. In addition, the EQ-5D scale has a large ceiling effect [22], so the EQ-5D scale cannot accurately reflect the health status of patients with hypertension.

Moreover, MMD patients take multiple drugs simultaneously, with serious economic burdens caused by drug costs. According to statistics, the direct economic cost to older adult patients caused by improper management of polypharmacy is as high as USD 2 billion annually [10]. In this study, the average daily medication therapy cost for patients at the initial admission phase was as high as RMB 137.16/day. After a 12-month follow-up, the average daily medication therapy cost for the intervention group decreased from RMB 50.58 to RMB 35.43, with a maximum decrease of RMB 76.03/day. The average daily medication therapy cost for the intervention group in the first, third, sixth, and twelfth months after discharge statistically decreased compared with that at the initial phase of admission (*P* < 0.05). The average daily medication therapy cost for the intervention group in the twelfth month after discharge significantly decreased compared with that of the control group (*P* < 0.05). According to a retrospective study, during a ten-year period, for 9068 patients who received MTM service, the total expenses of the health system decreased by USD 2,913,850 [23]. Briefly, compared with pharmacists’ general medication education and consultation, the MTM service could further reduce the economic burden on patients’ families, which could be regarded as one of the key tasks of pharmacists.

Studies have shown that patients with polypharmacy are more prone to problems such as improper medication management and adverse drug reactions, which increases hospitalization rates [24]. In this study, the readmission rate in the intervention group in the sixth and twelfth months after discharge was less than that in the control group (*P* > 0.05). This may be because the follow-up time is not long enough to reflect MTM’s advantage in reducing the readmission rate.

DRPs refer to events or situations in medication therapy that have interfered or will interfere with the expected therapy results. These problems include adverse drug reactions, medication errors, unclear purpose of drug use, and inappropriate drug selection. The incidence of DRPs gradually increases with the increase in the number of drugs used by patients; 79.8% of older adult patients with polypharmacy have at least one DRP [25], and adding another drug will increase the DRPs by 10% [26]. According to the studies, the incidence of DRPs in patients taking five and over ten types of drugs is 30% and 47%, respectively [10]. Many studies have shown that DRPs may lead to a decline in patients’ quality of life, an overall increase in hospitalization and medical costs, and even an increase in readmission rate and mortality [10, 27–29]. After a 12-month follow-up, the number of DRPs in the two groups all decreased. Moreover, the number of DRPs solved in the intervention group in the third, sixth and twelfth months after discharge were statistically higher compared with that in the control group. Drug use in MMD patients is characterized by a wide variety of drugs, complex usage and dosage, and long-term medication, which are critical reasons for poor patient compliance. Specifically, the most common situations include forgetting to take drugs, adverse drug reactions, and thinking they do not need to take the drugs [30]. This results in problems such as disease aggravation and deteriorating health conditions. After the MTM service had been given by Hale et al. [31] to the patients, the patient compliance significantly increased. Futhermore, Zhao et al. [32] confirmed that MTM services were efficacious in resolving DRPs and improving adverse drug reactions. They are consistent with our conclusion. Compared with general pharmaceutical services, MTM service could effectively solve these DRPs, reduce their adverse reactions, improve patient compliance and the accuracy of drug use in patients. This, in turn, would improve the patients’ quality of life, consistent with the results in the U.S. “Fairview Health Services” project [33].

It has been over ten years since MTM was proposed and has become a mature pharmaceutical care covered by Medicare in the United States. The effect of its implementation has also been tested in practice and confirmed by relevant research, and the effect is very significant in clinical, economic, and humanistic aspects.

The new “patient-centered” pharmaceutical care service model—MTM service—enables pharmacists with professional skills to help patients identify and solve problems in medication. Also, clinical pharmacists can be more quickly and better integrated into the clinical treatment team, which reflects the value of pharmacists and contributes to the development of hospital pharmacy in China [34, 35].

The MTM model will be established for MMD in patients with hypertension to help them understand their therapeutic drugs, reduce DRPs, improve patient compliance, reduce their financial burden, and actively convey health consciousness and a healthy lifestyle to patients. Meanwhile, pharmacists will be assisted in improving their pharmaceutical service and MMD management capability, strengthening their communication with patients, improving their service enthusiasm, and reflecting on their professional value.

In the future, a drug management model for chronic disease patients should be explored through medical associations, led by clinical pharmacists and participated in by pharmacists in community pharmacies, to obtain better service effects.

### Strengths and limitations

Strengths: The MTM mode was established for MMD inpatients. The evaluation indexes include clinical indicators, economic outcomes, human outcomes, and the number of DRPs. The evaluation is relatively comprehensive. Meanwhile, the pharmacists will be assisted in improving their pharmaceutical service and MMD management capability, strengthening their communication with patients, improving their service enthusiasm, and reflecting on their professional value.

Limitations: Clinical pharmacists delivered the intervention and assessed the outcomes, increasing the risk of biased assessment. However, the outcome was assessed using an objective measurement tool, which may have minimized the potential effects of this bias on the outcome. In the future, using third-party personnel to measure the clinical outcomes may help mitigate bias. Additionally, the overall drop-out rate in this study was 15.8%, which is slightly higher than that observed in previous pharmacy studies of patients. Due to the limited pharmacists in medical institutions, there are not enough pharmacists capable of undertaking pharmaceutical care. Older adults do not fully recognize MTMs, and long-term follow-up leads to a slightly higher rate of loss to follow-up. In the future, we will actively cooperate with community pharmacists to provide more comprehensive and long-term services to MMD patients. Finally, analyses were conducted and it was determined that the group lost to follow-up was not statistically associated with the group successfully followed up. While these analyses reduced selection bias to some extent, they may inevitably lead to biased results, for example, data on people who were missed due to deteriorating health status may have had some impact on the outcome of the intervention group. Therefore, we need larger sample sizes and better designs in the future.

## Conclusions

Patients with multiple NCDs often use many drugs simultaneously and need to take drugs for an extended period. However, unreasonable, or unsafe polypharmacy causes hospitalization, medication errors, and adverse reactions [11]. Therefore, the patients’ quality of life will be seriously affected, and health care resource expenditure will be significantly impacted. In this study, clinical pharmacists cared for the patients through the MTM service. Consequently, blood pressure was better controlled, and the incidence of DRPs was reduced. Further, the medication therapy effect was improved, patients were helped in controlling their illness, and their economic burden was simultaneously reduced. However, this effect is more obvious in long-term pharmaceutical care. Therefore, clinical pharmacists must adopt the MTM service for MMD patients, which helps ensure the safety and rationality of drug use in MMD patients.

## Supplementary Information

**Additional file 1.**

## Data Availability

The data and the trial protocol that support the findings in this study can be made available through contacting the corresponding author under reasonable request or in the link “http://www.medresman.org.cn/uc/projectsh/projectedit.aspx?proj=4897”.

## Abbreviations

DRP: Drug-related problem
MTR: Medication therapy review
NCD: Noninfectious chronic disease
HIS: The information system of Hospital
MMD: Multi-morbidity
PASS: Power Analysis and Sample Size
