# Supplementary material for: Impact of medication therapy management (MTM) service model on multi-morbidity (MMD) patients with hypertension: a pilot RCT
Source: BMC Geriatr. 2023 Jan 6;23:10. doi: 10.1186/s12877-023-03725-4 (PMC9824935; doi:10.1186/s12877-023-03725-4)
Supplement: Supplementary file 1 — Additional file 1. [file 12877_2023_3725_MOESM1_ESM.docx]

**Table S1. EQ-5D Utility Value Conversion Table**

| Dimension | Level | | | | |
| --- | --- | --- | --- | --- | --- |
|  | No problems | Slight problems | Moderate problems | Severe problems | Extreme problems |
| Mobility | 0 | 0.066 | 0.158 | 0.287 | 0.345 |
| Self-care | 0 | 0.048 | 0.116 | 0.21 | 0.253 |
| Usual activities | 0 | 0.045 | 0.107 | 0.194 | 0.233 |
| Pain/discomfort | 0 | 0.058 | 0.138 | 0.252 | 0.302 |
| Anxiety/depression | 0 | 0.049 | 0.118 | 0.215 | 0.258 |

EQ-5D, European Quality of Life Five Dimension

**Table S2. DRPs Classification in PAS System**

| Indication | Drug without indication | No indication |
| --- | --- | --- |
|  |  | Repetition of therapy |
|  |  | Non-medication therapy may be suggested |
|  |  | Adverse reactions may be avoided through therapy |
|  |  | Addiction/social medication |
|  | Untreated indication | There is an untreated condition or disease |
|  |  | Preventive medication therapy should be given |
|  |  | Another drug should be combined to enhance the curative effect. |
| Effectiveness | Different drugs need to be selected | There are more effective drugs |
|  |  | There are treatment contraindications |
|  |  | Inappropriate drug formulation |
|  |  | Ineffective disease treatment |
|  | Underdose | Wrong dosage |
|  |  | Inappropriate dosage frequency |
|  |  | Drug interaction |
|  |  | Inappropriate course of treatment |
| Safety | Adverse drug reaction | Undesirable pharmacological effects |
|  |  | Unsafe drugs to patients |
|  |  | Drug interaction |
|  |  | Excessively fast dosage adjustment speed |
|  |  | Allergic reaction |
|  |  | Drug contraindications |
|  | Overdose | Wrong dosage |
|  |  | Inappropriate dosage frequency |
|  |  | Inappropriate course of treatment |
|  |  | Drug interaction |
|  |  | Wrong dosage |
| Compliance | Non-adherence | Lack of understanding of drug instructions |
|  |  | Patient's unwillingness to take medicine |
|  |  | Patient's forgetfulness to take medication |
|  |  | Excessively expensive drugs |
|  |  | Unable to swallow/take medicine |
|  |  | Drug shortage |

DRPs, drug-related problems
